# Controversial Issues on EEG after Sleep Deprivation for the Diagnosis of Epilepsy

**DOI:** 10.1155/2013/614685

**Published:** 2013-06-12

**Authors:** Filippo Sean Giorgi, Michelangelo Maestri, Melania Guida, Elisa Di Coscio, Luca Carnicelli, Daria Perini, Chiara Pizzanelli, Alfonso Iudice, Enrica Bonanni

**Affiliations:** ^1^Section of Neurology, Department of Clinical and Experimental Medicine, University of Pisa, I56126 Pisa, Italy; ^2^Neurology Unit of the Department of Neuroscience, AOUP, I56126 Pisa, Italy; ^3^Sleep & Epilepsy Center, Neurocenter of the Civic Hospital (EOC) of Lugano, 6900 Lugano, Switzerland

## Abstract

EEG after sleep deprivation (SD-EEG) is widely used in many epilepsy centers as an important tool in the epilepsy diagnosis process. However, after more than 40 years of use, there are a number of issues which still need to be clarified concerning its features and role. In particular, the many scientific papers addressing its role in epilepsy diagnosis often differ remarkably from each other in terms of the type of patients assessed, their description and study design. Furthermore, also the length and the type of EEG performed after SD, as well as the length of SD itself, vary dramatically from one study to another. In this paper we shortly underscore the abovementioned differences among the different reports, as well as some interpretations of the findings obtained in the different studies. This analysis emphasizes, if needed, how SD-EEG still represents a crucial step in epilepsy diagnosis, and how additional, controlled studies might further shape its precise diagnostic/prognostic role.

## 1. Introduction 

The relation between sleep and epilepsy had already been described in scientific papers still before the use of EEG [1], and the role of sleep deprivation (SD) in promoting epileptic seizures and facilitating interictal epileptiform abnormalities (IIAs) has been studied since the 60s [2]. 

EEG after sleep deprivation (SD-EEG) was thus proposed as a method to increase the yield of EEG in revealing IIAs in patients with suspected seizures and to further improve the accuracy of the diagnosis of epilepsy. Several experimental studies in animal models, healthy controls, and epileptic patients highlight the role of SD and sleeping during an inappropriate circadian phase (i.e., in the morning) in enhancing sleep instability and possibly causing the occurrence of IIAs [3]. Moreover, SD enhances cortical excitability in patients with different subtypes of epilepsy more than in controls [4]. These pathophysiological issues are beyond the aim of this paper, in which we would rather focus on several controversial issues that make the interpretation of findings obtained by SD-EEG difficult even after more than 40 years of its use. 

## 2. Variability in SD-EEG Protocol and Examined Population 

The results of published SD-EEG studies testing this method are difficult to compare to each other, mainly because of the protocols used and the population of patients assessed. Concerning the first issue, the protocol of SD (total or partial), the length of the SD-EEG recording, the recording of drug-induced sleep, and the time of the day of the recording (morning or afternoon) constitute the main variables. Moreover, the inclusion and exclusion criteria in the published papers are very different concerning age (children versus adults), seizure and epilepsy types, absence of abnormalities on basal EEG, neuroimaging, and treatment with antiepileptic drugs (AEDs). Again, even the definition of epileptic IIAs is not homogenous and, last but not least, most of the studies are retrospective, using Epilepsy Center databases. 

*The protocol of SD* (i.e., SD lasting at least 24 h, or partial SD) varies significantly in the different studies. In most of the earlier reports, the authors induced at least 24 h SD, while, more recently, several groups started testing the effects of partial SD (see, e.g., [5–9]) with lengths of sleep allowed during the night before SD-EEG varying from 3 h [8] up to 7 h [6]. 

Interestingly, almost unanimously in studies on children, authors use age-related partial SD, increasing with patients' age [5, 6, 8, 10–15], and since such duration also varies significantly from one study to another, this represents another confounding factor for interpreting its role for epilepsy diagnosis. 

Also, *the duration of EEG recordings*, which is a potentially critical issue for IIAs yield (see below), varies significantly among different centers, ranging from 30 minutes [16] up to even 24 h [17]; further, in some studies authors include, during SD-EEG recordings, also hyperventilation and intermittent photic stimulation, while others do not (see below). 

The *time of the day of the recording* (morning, afternoon, or night) is related to different circadian rhythms and mechanisms of sleep (see as a review [18]) and possibly to a different risk of occurrence of IIAs in different types of epilepsy. 

Apart from basal EEG and SD-EEG, in the same study, some authors performed also a further EEG during *sleep induced by hypnotic drugs*, such as promazine, barbiturates, or benzodiazepines [19–22]. 

The last issue to be considered concerning SD protocol is the *interval* between the last suspected seizure and the SD-EEG recording. A residual postictal activation seems to be likely only when performing SD-EEG within 2-3 days after seizure [6, 23], but in the routine clinical practice this rarely occurs, and thus it is not as important as expected [9]. 

Another major issue varying from one study to another is the *population* studied. Even though the majority of the studies recruited patients undergoing a complete evaluation for suspected epilepsy, other inclusion and exclusion criteria are often not comparable, and in many cases it is not even possible to separate and analyze single variables. 

In fact, only few prospective series have been published [11, 14, 22, 24–27], while most studies are retrospective using data from Epilepsy Center databases. 

Even though age is considered critical for SD-EEG outcome, in some reports both adults and children have been included in the same group [5, 6, 8, 10, 16, 21, 24, 25, 28–34]. Moreover, although occurrence of *IIA during the first routine wake EEG* could be considered as an exclusion criterion in studies testing SD-EEG sensitivity, this aspect is sometimes not evaluated or not considered as a bias (see, e.g., [6, 10, 11, 15, 16, 20, 29, 33, 35]). 

Concerning the *classification of epilepsy*, the oldest studies included patients with different types of seizure or syndrome, which were often only roughly classified [6, 10, 22, 24, 25, 28, 31, 32, 34, 36–39], while some of the newer ones included populations more homogeneous concerning those aspects [16, 17, 40–44]. In some papers, epilepsy/seizures classification and other clinical features are not even clearly specified (see [5, 7, 8, 11, 21, 33, 45, 46]). 

*Therapy* with AEDs is another aspect varying from one study to another. Only in a few studies SD-EEGs were performed in *de novo *patients which had never been treated with AEDs [6, 8, 9, 14, 20, 23], while in most of the remaining ones, also patients taking AEDs were included, and therapy was left unchanged or at least AEDs tapering was performed thus probably significantly affecting occurrence of IIA and, thus, sensitivity and specificity of SD-EEG [47–49]. Furthermore, the number and type of AEDs were not described in detail in most papers (see, e.g., [10, 11, 13, 15, 16, 22, 24, 25, 27, 31, 32, 34, 40, 42, 44]), with some exceptions [17, 41, 46]. 

Another critical issue is the *definition of IIAs,* since usually EEG activation was defined by the occurrence of specific epileptic IIAs, but in some studies the authors considered also occurrence of slow waves [11, 12, 20, 23, 29], and, in a surprisingly high number of studies, the types of EEG abnormalities were not even specified [13, 21, 26, 32, 33, 38, 39]. 

Several epilepsy syndromes (especially IGE, but also some focal ones) often show a photoparoxysmal/photoconvulsive response to intermittent light stimulation performed during EEG. The potentiation/unveiling of such an effect might occur after SD thus significantly helping in the diagnosis. Unfortunately, even in the few SD-EEG studies including such a protocol, a detailed analysis of the results was not reported, or, when IIAs occurrence during photic stimulation was listed, a correlation with seizure type(s) or syndrome was not provided (see, e.g., [21, 22, 37]).

## 3. Interpreting the Role of SD-EEG in Epilepsy: Does SD Effect Exist? 

Two important questions about the role of SD in inducing IIAs and thus in the diagnostics process of epilepsy are (1) whether the increased sensitivity of SD-EEG is due just to an effect of sampling or length of the recording, and (2) whether sleep *per se *or rather SD, indeed, induces activation of the EEG. 

Concerning the first issue, some authors hypothesized that the occurrence of IIAs during SD-EEG is due just to a sampling effect related to a second EEG [22], since it is known that the sensitivity of EEG increases proportionally to the number of repeated routine EEGs [50–52]. However, the papers analyzing the role of a second routine EEG in patients with IIAs during SD-EEG do not support this hypothesis. 

Three studies, back in the 60s, showed that only a very small percentage of patients (<20%) with IIAs during SD protocol presented also an abnormal second routine EEG [24, 25, 36]. Recently, our group found retrospectively that, among 61 epileptic patients bearing a basal normal/nonspecific EEG, a second routine EEG revealed IIAs in 13.1%, while IIAs occurred in 45.9% of their SD-EEG [9]. In an elegant prospective study [20], SD-EEG was more likely to show epileptiform discharges as compared to routine EEG, SD-EEG, and drug-induced sleep EEG, performed in random order. 

The duration of SD-EEG recording should be also considered, since SD-EEG recording lasts usually much longer than basal EEG. Even though there is no formal study on this issue, in the general experience, IIAs occur also during the first part of SD-EEG and the sensitivity of SD-EEG is similar in studies using different durations of recordings [9, 39]. 

Another debated aspect is whether sleep *per se* or SD induces EEG activation. Apart from physiological speculations, current data have to be considered as not definitive, and the provocative effects of both SD and sleep/drowsiness are likely to enhance each other. 

Some data suggested that EEG activation is already present during the waking phases of EEG recorded after SD [15, 24, 25, 30–32, 37, 40, 42]. However, in most cases, epileptiform discharges occurred more frequently during sleep [11, 14, 46], in particular during light sleep stages [8, 9, 19, 22, 23]. Some exceptions exist in which IIAs occurred both during wakefulness and sleep [5, 34, 35, 37]. 

When directly compared to each other, spontaneous sleep seemed to increase significantly generalized discharges, while sleep occurring after SD might increase more focal discharges [39]. On the other hand, the comparison between SD-induced sleep and drug-induced sleep gave discordant results. Four studies [16, 32, 35, 40] showed a similar yield in IIAs occurrence between the two approaches, while the other three ones [20–22] found a significantly higher activation rate in SD-EEG. 

## 4. Sensitivity and Specificity of SD-EEG in Epilepsy and in Different Syndromes 

Given the dramatic differences highlighted above, the variability of sensitivity and specificity in single studies is not surprising. In fact, the occurrence of IIAs during SD-EEG ranges from 20% [42] to 57% [45] in adult patients with the diagnosis of epilepsy, and between 32% [36] and 54% [30] in children. 

Some features of epilepsy seem to be more likely to be associated with IIAs occurrence during SD: activation seems to be greater shortly after epilepsy onset or seizures occurrence [23, 25, 31, 40], in patients with an earlier seizure onset [19], and in those with a history of recurrent seizures [15, 23]. 

Concerning the role of different types of syndrome or seizures, the first observation, by Mattson et al. [24], reported a slightly higher activation rate in patients with “grand mal” seizures, than in those with “psychomotor” seizures, and few years later, Pratt et al. [25] found SD EEG activation in 41% of patients with grand mal seizures, 47% of psychomotor seizures, and 37% of other focal seizures. The main limit of these studies was the lack, at that time, of an unanimously accepted seizure classification. In more recent observations, even though data are not consistent and numerous, we could state that, according to R. Degen and H.-E. Degen [16], activation is more frequent in patients with complex partial seizures only, as compared to complex partial seizures plus other seizures types. Gandelman-Marton and Theitler [23] did not find any activation in patients with focal seizures, while 29% of patients with focal seizures with secondary generalization and 20% of patients with primary generalized tonic-clonic seizures presented IIAs during SD. Concerning syndrome classification, a higher activation in idiopathic generalized epilepsy [9, 11] and in particular in awakening grand mal and childhood absence epilepsy [19, 32], has been reported. 

Neuroimaging data were available for few patients only in few recent SD EEG casistics [6, 9, 10, 17, 23, 27, 39, 41, 44]. In most of them, the number of patients included was too low for allowing statistical correlations between specific size/site/nature of the lesions and neurophysiological data. Also, in our recent study [9], the diagnostic power for SD-EEG is not statistically different among subgroups of focal epilepsies. In the largest study [6] in 300 *de novo* patients, among which only those with a previous negative basal EEG underwent a SD-EEG, the authors found that 17% of patients with EEG diagnosis of partial epilepsy had abnormal CT/MRI. 

Since the earliest studies on SD EEG, specificity has been assessed and shown to be very high. Back in the late 60s, two papers showed a specificity of 99 and 100% respectively [24, 36], and more recent papers showed similar results, thus confirming an occurrence of IIAs in 0 up to 12% of adult controls [27, 35]. Even more recently, in a retrospective study assessing the role of partial SD EEG in a wide population of patients assessed for suspected seizure, bearing a normal basal EEG and with a prolonged followup, we confirmed a high specificity rate (91.1%) [9].

## 5. Conclusions and Future Perspectives 

Epilepsy is a complex disease, whose diagnosis is the results of the combination of anamnesis data and clinical history with diagnostic techniques, among which neuroimaging and EEG play a pivotal role. Actually, it is more appropriate to talk about epilepsies, rather than epilepsy, because different syndromes/seizure types differ markedly from each other, in terms of aetiology, pathophysiology, prognosis, and of appropriate treatment. An “ideal” diagnostic tool should be able to discriminate between epilepsy and non-epilepsy, that is, it should help to predict the likelihood of seizure recurrence in subjects experiencing a first seizure. The predictive value of the diagnostic exam is particularly crucial for epilepsy, especially in light of the burden of potential side effects of AEDs, which often need to be taken for years by patients, and of the stigma still surrounding the diagnosis of epilepsy. Thus, such a test should be very specific for epilepsy, and as sensitive as possible, in order to avoid the potential risk of not treating epileptic. As described above, SD-EEG has been generally shown to bear a high specificity, and seems to be, thus, a good diagnostic tool. 

However, in the previous paragraphs we underscored the main difficulties in getting the full-blown potentiality of SD-EEG recording: these are mainly related to the huge methodological variability among the different studies in the field. Among them, the most relevant ones are represented by the striking differences in patients population and the SD-EEG protocols themselves, which often varies significantly from one centre to another. Furthermore, many of the most important studies on SD-EEG and epilepsy date back to several decades ago, when neuroimaging data on the patients were lacking (especially the nowadays routinely performed MRI data) and some epilepsy syndromes subtypes had not been detailed yet. 

An “ideal study” to clarify most of the abovementioned issues should include at least: (a) *de novo* potentially epileptic patients, in order to rule out the potential effects of AEDs on SD-EEG sensitivity; (b) data concerning multiple routine EEGs and SD-EEG for each patient, in order to test directly the role of the sampling effect on the diagnostic yield of SD-EEG; (c) a comparison of different lengths of SD and EEG recording, in order to help selecting the protocol with the highest potential compliance; (d) for each EEG recording (either basal or after SD) the occurrence of IIAs during the wake period, in order to clarify the role of sleep *per se* versus a specific role of SD; (e) performing additional stimulation, such as intermittent light stimulation, to address the effect of SD on its IIAs yield; (f) for each patient, an adequate followup in order to detail the occurrence of epileptic seizures, that is, the diagnosis of epilepsy. Of course, a prospective approach and an adequate amount of patients would be preferable; in any case, the detailed analysis of EEG IIAs occurrence should be performed by investigators rigidly blinded to the final diagnosis (i.e., epilepsy or not epilepsy) and features of the patients. The latter is a key requisite for avoiding the potential bias of over interpreting the role of a technique which, by itself, is considered crucial for the diagnosis itself, and is difficult to be ruled out in most of the existing studies on SD-EEG, which are retrospective in nature. 

A study bearing all of the features as above is, of course, impossible to be performed. However, it would be already important to have studies fulfilling at least some of the abovequoted features; these should be multicenter to recruit as many patients as possible. 

In conclusion, the history of the role of SD-EEG in epilepsy is still far from being fully elucidated, and many results suggest that this approach should not be considered *âgée*, but rather still one of the most useful diagnostic tools in the hands of epileptologists for many more years.
